# Trace element accumulation in hooded crow nestlings: tissue- and age-specific patterns and implications for avian biomonitoring in riverine wetlands

**DOI:** 10.1007/s10661-026-15807-y

**Published:** 2026-08-14

**Authors:** Piotr Zduniak, Jakub Z. Kosicki, Izabela Komorowicz

**Affiliations:** 1 Department of Avian Biology and Ecology, Faculty of Biology, Adam Mickiewicz University, Poznań, Uniwersytetu Poznańskiego 6, Poznań, 61-614 Poland; 2 Department of Trace Analysis, Faculty of Chemistry, Adam Mickiewicz University, Poznań, Uniwersytetu Poznańskiego 8, Poznań, 61-614 Poland

**Keywords:** Biomonitoring, Blood, Feathers, *Corvus cornix*, Nestlings, Non-essential elements

## Abstract

Floodplain wetlands are dynamic ecosystems that may receive pollutants transported from upstream areas, but local exposure of wildlife to trace elements can vary depending on hydrology, bioavailability, habitat structure, and diet. In this study, we assessed the concentrations of potentially toxic non-essential elements (Cd, Pb, As) and essential elements (Cu, Se, Zn) in the blood and feathers of hooded crow (*Corvus cornix*) nestlings from a riverine wetland in Warta Mouth National Park, western Poland. Samples were collected at two developmental stages and analyzed using inductively coupled plasma mass spectrometry (ICP-MS). Concentrations of Cd, Pb, and As were consistently low in both tissues, suggesting limited exposure and/or low bioavailability of these elements in the local food web used by this omnivorous wetland-associated species. Element concentrations varied with nestling age. Older nestlings had higher blood Cu and Se concentrations. In feathers, Cd and Cu concentrations were also higher in older nestlings, whereas Zn concentrations were lower than at the earlier growth stage. These age-related differences may reflect a combination of physiological regulation, growth requirements, tissue-specific deposition, feather development, and element-specific toxicokinetics. No significant relationships were found between blood and feather concentrations, indicating that these matrices provide complementary information on exposure during nestling development. Feather Zn concentration was positively associated with nestling body condition, whereas no such relationship was detected for potentially toxic non-essential elements. Our findings highlight the importance of considering tissue type, feather ontogeny, and nestling growth stage when interpreting trace element concentrations in avian biomonitoring.

## Introduction

Human activities release a wide range of pollutants into the environment, including trace elements that may be toxic to living organisms when present at elevated concentrations (Briffa et al., 2020; Kabata-Pendias, 2010; Kolarova & Napiórkowski, 2021; Mikulewicz et al., 2017). Because trace elements can persist in soils, sediments, and aquatic systems, their occurrence and distribution are widely used to assess environmental contamination associated with agriculture, industrialization, urbanization, and other forms of human pressure (Boyd, 2010; Hayzoun et al., 2014; Morin-Crini et al., 2022).

The degree of contamination in a given area depends on local and regional factors affecting the quality of air, water, soil, and sediments. Elevated concentrations of trace elements may result from proximity to pollution sources such as mining areas, industrial infrastructure, wastewater discharge, agricultural runoff, and urbanized areas (Chatelain et al., 2021; Dusengemungu et al., 2022; Long et al., 2021). Pollutants can also be transported over long distances from their emission sources by air masses or through river systems, leading to contamination in areas located far from the source (Krodkiewska et al., 2022; Roiger et al., 2012).

Freshwater floodplains are particularly relevant for studies of trace element exposure because they are dynamic transitional zones between river channels and terrestrial habitats (Mitsch & Gosselink, 2000; Tockner & Stanford, 2002). During flooding events, river water may transport suspended particles, organic matter, nutrients, and contaminants onto floodplain surfaces, where sedimentation can lead to local deposition of pollutants (Baborowski et al., 2007; Ferreira et al., 2023; Haarstad et al., 2012). At the same time, floodplain wetlands are highly heterogeneous and hydrologically dynamic systems. Processes such as dilution, sediment redistribution, flushing, and changes in element bioavailability may strongly influence the actual exposure of organisms inhabiting these areas (Diehl et al., 2023; Dordio et al., 2008; Thonon, 2006). Floodplain wetlands are also ecologically important ecosystems and are often recognized as biodiversity hotspots (Holgerson et al., 2019; Mitsch & Gosselink, 2000; Tockner & Stanford, 2002). Their complex habitat structure supports diverse food webs, including aquatic, semi-aquatic, and terrestrial organisms. However, the same hydrological and trophic connectivity that supports high biodiversity may also create pathways for contaminant transfer through the environment and food web (Bjedov et al., 2024; López-Perea et al., 2019). Among wetland contaminants, metals and metalloids are of particular concern because of their potential toxicity and capacity to enter biological tissues through exposure, uptake, and retention processes (Bjedov et al., 2024; López-Perea et al., 2019).

Birds are widely used in biomonitoring studies because many species are relatively easy to observe and sample, occupy different trophic positions, and may reflect environmental contamination through element concentrations in their tissues (Hanć et al., 2017; López-Perea et al., 2019; Movalli et al., 2017). Although internal tissues can provide valuable information on contaminant accumulation, their collection often requires invasive or lethal sampling (e.g., Binkowski et al., 2013; Nam et al., 2005). Therefore, blood and feather samples are frequently used as less invasive matrices in avian ecotoxicology (Bjedov et al., 2024; Dolan et al., 2017). The blood usually reflects recent or current exposure, whereas feathers can integrate exposure during feather growth and may also reflect tissue-specific deposition or excretion of elements (Burger, 1993; Jaspers et al., 2019).

In this study, we investigated trace element concentrations in hooded crow (*Corvus cornix*) nestlings from a riverine wetland located within Warta Mouth National Park, western Poland. The study area is situated at the mouth of a large lowland river and may receive pollutants transported from upstream parts of the catchment. The hooded crow is an omnivorous and opportunistic corvid whose diet in wetland habitats may include prey and food items from aquatic, semi-aquatic, and terrestrial sources. This makes the species a useful sentinel for assessing local exposure to trace elements across multiple trophic pathways, although it should not be considered equivalent to specialized avian predators or apex predators. The goals of the study were (1) to assess the occurrence of selected potentially toxic non-essential elements (Cd, Pb, As) and essential elements (Cu, Se, Zn) in the blood and feathers of hooded crow nestlings; (2) to determine whether the concentrations change with the age of the nestlings; (3) to compare the concentrations between blood and feather matrices, and (4) to assess whether element concentrations are associated with nestling body condition. By combining repeated sampling of individually marked nestlings with analyses of two biological matrices, this study contributes to the biomonitoring of trace element exposure in a protected floodplain wetland and helps clarify how tissue type and developmental stage influence the interpretation of element concentrations in nestling birds.

## Materials and methods

### Study species

The hooded crow (*Corvus cornix*) is a socially monogamous, solitary-nesting corvid widely distributed in the western Palaearctic (Goodwin, 1976). It is an omnivorous and opportunistic species that uses a broad range of food resources, including animal prey, carrion, plant material, and anthropogenic food sources, depending on local habitat conditions and food availability. In wetland landscapes, hooded crows may forage in aquatic, semi-aquatic, and terrestrial habitats and may include fish, molluscs, crayfish, amphibians, eggs, nestlings, and other animal material in the diet of their young (Zduniak et al., 2008). In the study area, the species reaches a density of 2.6–3.8 breeding pairs per square kilometer (Zduniak, 2006). Its broad trophic niche and use of food items from multiple habitat compartments make it a useful sentinel species for assessing local trace element exposure in floodplain wetlands. However, because hooded crows are omnivorous generalists, they should not be interpreted as equivalent to specialized avian predators or apex predators.

### Study area

**Fig. 1:**
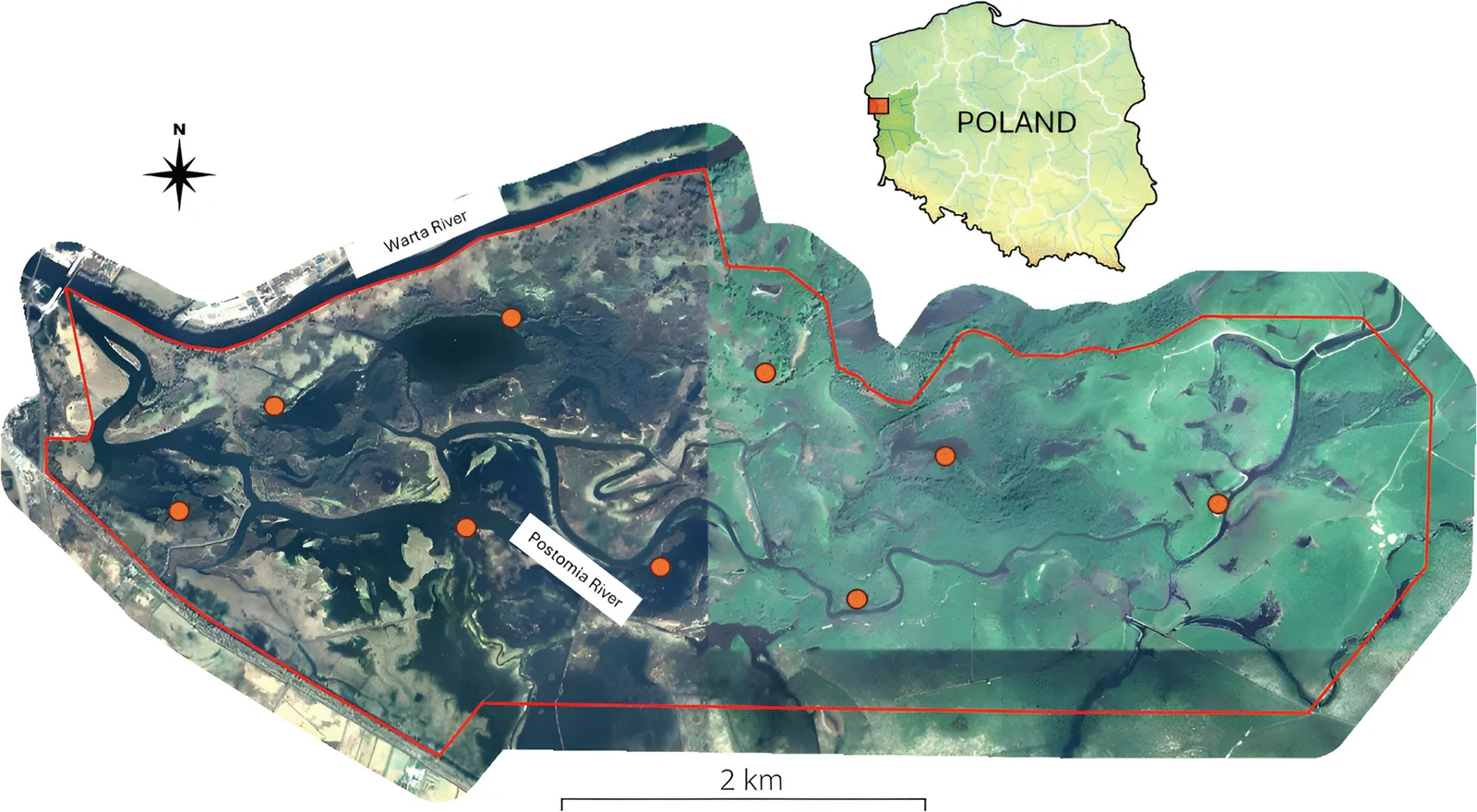
Map of the study area, with the boundary of the study plot marked in red and the locations of studied broods in orange. The basemap is sourced from Google Earth

The study was conducted in Warta Mouth National Park (52°34’N, 14°43’E; Fig. 1), which is of international importance as a wildfowl refuge (Grimmet & Jones, 1989) and is protected under the Ramsar Convention.

The Park is located in western Poland at the confluence of the Warta and Odra rivers. As a result of land reclamation efforts completed in the early twentieth century, the main channels of both the Warta and Odra rivers were embanked, and much of the floodplain was cleared of trees. On the left bank of the Warta, just before its confluence with the Odra, a retention reservoir of approximately 51 km^2^ was created. This reservoir buffers seasonal water level fluctuations in the Warta River. Consequently, most of the Park, located within this retention area, is characterized by dynamic hydrological conditions and low long-term repeatability of water levels (Choiński, 2000; Zduniak, 2009).

The Warta River, the third-longest river in Poland, is 808 km long and drains a catchment area of approximately 54,000 km^2^. This basin includes intensively cultivated farmland and several major urban centres, including Poznań, Konin, and Gorzów Wielkopolski. As a result, the river is exposed to multiple sources of pollution, such as municipal and industrial wastewater, agricultural runoff, and urban contaminants (Jaskuła et al., 2021). According to regional environmental assessments, there are no sections of the Warta River where surface water quality meets the criteria for “good” status (Marshal’s Office of Lubuskie Voivodeship, 2017). Previous studies have indicated that the terminal section of the Warta River, within the boundaries of Warta Mouth National Park, may be affected by pollutants transported from upstream parts of the catchment, including metals and biogenic compounds (Czyż et al., 2013; Lanocha et al., 2014). Another river flowing through the studied area is the Postomia River, which is 67 km long and has a catchment area of 1425 km^2^. In contrast to the Warta, the Postomia River is considered relatively unpolluted. Concentrations of pollutants, including metals and rare earth elements (REEs), in its headwaters are low, suggesting primarily geogenic sources (Siepak et al., 2023).

The study plot covered 16 km^2^ and was situated in the western part of the Park, entirely within the retention reservoir (Fig. 1). The area is dominated by a mosaic of herbaceous vegetation, primarily reed-canary grass (*Phalaris arundinacea*), together with arborescent vegetation consisting of old willow trees and shrubs (*Salix* sp.). The landscape also includes shallow lakes, former river channels, ditches, and dikes, contributing to its ecological and hydrological heterogeneity. All sampled nests were located within this plot, in willow trees growing in the floodplain mosaic described above. Thus, the samples were collected from nestlings inhabiting the western, reservoir-influenced part of the Park, where local exposure may be shaped by seasonal flooding, sediment redistribution, vegetation structure, and the availability of aquatic, semi-aquatic, and terrestrial food resources. The positions of sampled nests are shown in Fig. 1.

### Fieldwork

The fieldwork was conducted during the breeding season, from April to June 2014. Data on nest location and breeding phenology were collected during regular nest visits, following the procedures described by Zduniak (2009). Nestlings were sampled from nests located 8–15 m above ground level. Chicks were lowered from nests in a cotton bag using a rope, ringed, measured, sampled, and then returned to their nests.

All nestlings were measured by the same person. Total head length and tarsus length were measured to the nearest 0.1 mm using a sliding calliper, and body mass was measured to the nearest 5 g using a Pesola spring balance. Approximately 1.5 mL of the blood was collected from the brachial vein using a heparinized syringe. Blood samples were transferred into Eppendorf tubes and stored at − 20 °C until analysis. During each sampling event, one primary feather (P1) was collected. At the first sampling, P1 was collected from the left wing, whereas at the second sampling, P1 was collected from the right wing. In total, blood and feather samples were collected from 29 nestlings from 9 nests. Of these, 24 nestlings from 7 nests were sampled twice at an interval of 7–8 days, whereas 5 nestlings were sampled only once. The age of the nestlings ranged from 14 to 25 days. The first sampling was conducted when nestlings were 14–18 days old, and the second sampling when they were 22–25 days old. Feathers collected during the first and second sampling events were treated as separate samples and were digested and analyzed independently, not pooled before analysis. Thus, the sampling design yielded 53 blood samples and 53 feather samples.

### Feather and blood sample preparation

Feather samples were subjected to a cleaning process using an ultrasonic bath to remove surface contaminants. This involved multiple washings with a solution composed of distilled water and acetone. Subsequently, the feathers were rinsed in distilled water to remove residual acetone and then dried at 105 °C until a constant weight was achieved. Once desiccated, the samples were transferred directly into quartz digestion vessels. For digestion, a mixture of 65% (v/v) Suprapur nitric acid (Merck, Germany) and 30% (v/v) hydrogen peroxide (Stanlab, Poland) in a volumetric ratio of 2:1 was added to each sample and allowed to stand overnight at ambient temperature. On the following day, the quartz vessels were placed inside Teflon containers and processed in a microwave digestion system (Ethos One, Milestone, Italy). The microwave digestion protocol consisted of three sequential stages: (1) a 15-min power ramp to 1500 W, (2) a 30-min hold at 1500 W, and (3) a 30-min cooling step, with the digestion temperature maintained at 220 °C throughout. Post-digestion, the contents were quantitatively transferred to volumetric flasks and diluted to a final volume of 50 mL using ultrapure water. Procedural blanks containing the same reagent composition were prepared and digested alongside the samples in each digestion batch to monitor potential contamination.

For blood samples, the thawed material was transferred into 15 mL DigiTubes and digested directly with 2 mL of 65% (v/v) nitric acid (Merck, Germany) and 1 mL of 30% (v/v) hydrogen peroxide (Stanlab, Poland). The digestion process lasted for 3 h. Following digestion, each sample was diluted to a final volume of 40 mL before analytical measurements.

### Trace element analysis

Elemental concentrations were determined using an Agilent 7700 × inductively coupled plasma mass spectrometer (ICP-MS, Agilent Technologies, USA), equipped with a collision cell (CC) to minimize spectral interferences. The isotopes analyzed included ^63^Cu, ^66^Zn, ^75^As, ^82^Se, ^111^Cd, and ^208^Pb. Spectral interferences were mitigated through the application of collision cell technology, while non-spectral interferences were corrected using internal standards, specifically ^74^Ge, ^103^Rh, and ^159^ Tb.

### Analytical performance

Calibration curves were established using six concentration levels, ranging from 0.05 to 100 μg/L. All calibration curves exhibited correlation coefficients (*R*^2^) exceeding 0.999, indicating excellent linearity across the entire concentration range for all analyzed elements. To ensure analytical accuracy and data quality, certified reference materials (CRMs) were employed. CRM Human Hair NCS ZC81002B (China National Analysis Center for Iron and Steel, Beijing, China) was used to validate the measurements in feather samples, whereas Seronorm™ Trace Elements Whole Blood L-2 (Sero AS, Norway) served as the quality control material for blood sample analysis. The recovery rates for the elements determined in CRM Human Hair NCS ZC81002B were as follows: 89% for Cu, 94% for Zn, 102% for As, 120% for Se, 85% for Cd, and 99% for Pb. In turn, the recovery values calculated from results for Seronorm™ Trace Elements Whole Blood L-2 were 99% for Cu, 97% for Zn, 103% for As, 119% for Se, 94% for Cd, and 104% for Pb. The limits of detection for blood samples, calculated using calibration curve parameters, were determined to be: 0.018 μg/L for Cu, 0.12 μg/L for Zn, 0.0073 μg/L for As, 0.274 μg/L for Se, 0.0064 μg/L for Cd, and 0.0041 μg/L for Pb.

Precision was evaluated based on replicate measurements of the certified reference materials and expressed as the coefficient of variation (CV). The CV values for Human Hair NCS ZC81002B ranged from 1.5% for Zn to 6.9% for As. In the case of Seronorm™ Trace Elements Whole Blood L-2, precision values ranged from 1.4 to 3.0%, with the lowest variation observed for Se and the highest for As.

### Data processing and analysis

To assess nestling body condition, we calculated the scaled mass index (SMI) following the method proposed by Peig and Green (2009). This index allows for the comparison of body condition across individuals of different sizes by standardising body mass to a common body length. The SMI was calculated using the following equation: $SM{I}_{i}={M}_{i}{(\frac{{L}_{0}}{{L}_{i}})}^{{b}_{SMA}}$, where $SM{I}_{i}$ is the scaled mass index for individual *i*, ${M}_{i}$ is the body mass of individual *i*, ${L}_{i}$ is the tarsus length (used as a proxy for body size) of individual *i*, ${L}_{0}$ is the population mean tarsus length (used as the scaling reference), ${b}_{SMA}$ is the scaling exponent derived from a standardized major axis (SMA) regression of log-transformed body mass on log-transformed tarsus length. The value of ${b}_{SMA}$ was obtained from a log–log SMA regression performed on the entire sample population. The SMI provides a size-adjusted estimate of body condition that is more biologically meaningful than simple mass–length residuals, as it maintains the original mass units and allows for direct comparisons across individuals. Descriptive statistics for body mass, tarsus length, L₀, b_SMA_, and SMI values are reported in the Results section to ensure reproducibility of the SMI calculation. To document the morphometric basis of the body condition index, the body mass and tarsus length of nestlings used in the SMI calculation were summarized descriptively. Body mass ranged from 245 to 505 g, and tarsus length ranged from 44.3 to 61.5 mm. The population mean tarsus length used as the scaling reference was *L₀* = 55.4 mm, and the scaling exponent derived from the SMA regression was *b*_*SMA*_ = 1.869. The resulting SMI values ranged from 346.6 to 472.6.

Samples collected repeatedly from the same individuals were treated as dependent observations, as were samples from nestlings originating from the same nest. We assessed differences in element concentrations in blood and feather samples between the first and second sampling events using generalized linear mixed models (GLMMs) with a Gamma distribution and a logarithmic link function. Element concentrations served as response variables, sampling event (coded as 1 or 2) was included as a fixed effect, and nestling ID nested within nest ID was specified as a random effect. Additionally, we tested relationships between element concentrations in blood and feathers at each sampling event. In these models, the concentration of a given element in the blood was treated as the response variable, the corresponding feather concentration as the predictor, and nestling ID nested within nest ID was again included as a random effect. Furthermore, we tested whether element concentrations in the analyzed tissues were associated with nestling condition. In these GLMMs fitted with a normal distribution and an identity link function, nestling condition was the dependent variable, element concentrations in the blood and feathers were included as fixed effects, and nestling ID nested within nest ID was specified as a random effect. Two influential outliers located more than three standard deviations above the mean were removed from the analyses. All analyses were conducted using IBM SPSS Statistics for Windows (IBM Corp., 2023).

## Results

**Fig. 2:**
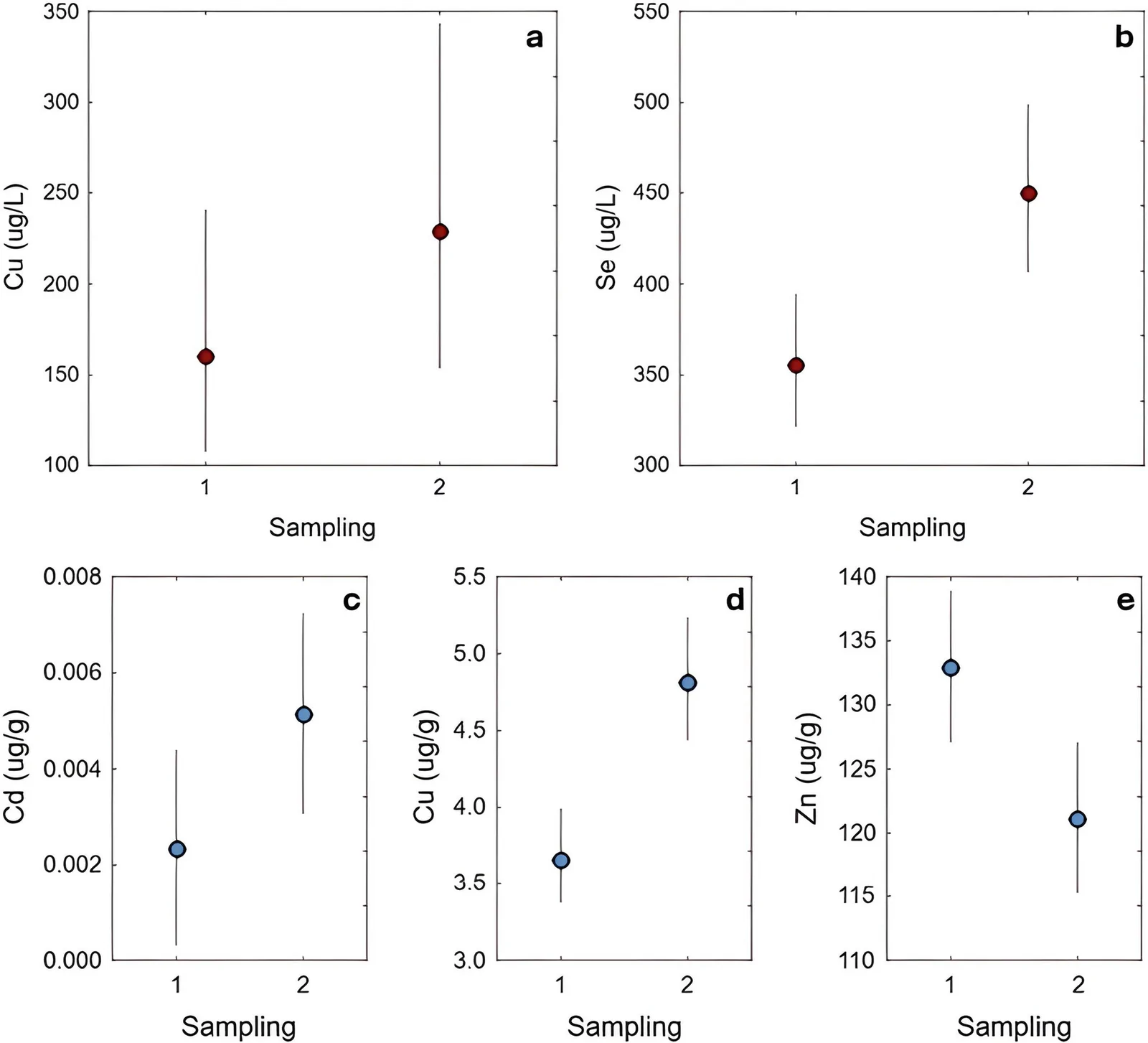
Element concentrations measured in the blood (**a**, **b**) and feathers (**c**–**e**) of hooded crow nestlings at two time points separated by 7–8 days; mean values are displayed with 95% confidence intervals

The obtained concentrations of individual elements in blood and feather samples are presented in Table 1. We observed differences in blood copper and selenium concentrations with higher levels at the older age compared to the earlier sampling (Fig. 2a, b); however, no age-related effect was observed for the other analyzed elements (Table 2).

**Table 1:** Descriptive statistics of element concentrations in hooded crow (*Corvus cornix*) nestlings’ blood (B; µg/L) and feathers (F; µg/g)

**Element**	**Sample**	**Mean (95% CL)**	**Median (Q25–Q75)**	Range
Cd	B	< LOD	< LOD	< LOD
	F	0.004 (0.002–0.007)	0.002 (0.000–0.006)	0–0.057
Pb	B	2.936 (1.883–3.989)	1.966 (0.977–3.387)	0–24.252
	F	0.139 (0.092–0.186)	0.081 (0.043–0.170)	0–0.893
As	B	9.251 (8.28–10.221)	7.877 (6.959–11.199)	3.826–20.437
	F	0.082 (0.068–0.095)	0.066 (0.055–0.084)	0.036–0.262
Cu	B	226.301 (195.373–257.229)	202.616 (172.136–248.358)	0–685.387
	F	4.514(3.976–5.052)	4.225 (3.545–4.738)	2.612–16.423
Se	B	398.646 (368.125–429.167)	394.302 (314.766–458.992)	127.878–656.916
	F	1.718 (1.645–1.791)	1.704 (1.559–1.843)	1.224–2.351
Zn	B	2194.263 (2043.279–2345.248)	2115.000 (1896.867–2340.806)	1188.513–4642.072
	F	126.773 (123.152–130.394)	126.709 (117.876–133.453)	98.207–167.426

**Table 2:** Summary of generalized linear mixed models (GLMMs) testing the effect of nestling age (comparison between samplings 1 and 2) on element concentrations in their blood and feathers

Sample	Element	*F*	DF	*p*
Blood	As	0.31	1, 44	0.582
	Cu	10.4	1, 44	0.002
	Pb	3.79	1, 43	0.058
	Se	16.96	1, 44	< 0.001
	Zn	1.66	1, 44	0.204
Feathers	As	0.91	1, 46	0.344
	Cd	7.08	1, 45	0.011
	Cu	39.54	1, 45	< 0.001
	Pb	0.49	1, 46	0.487
	Se	0.28	1, 46	0.599
	Zn	16.01	1, 46	< 0.001

**Fig. 3:**
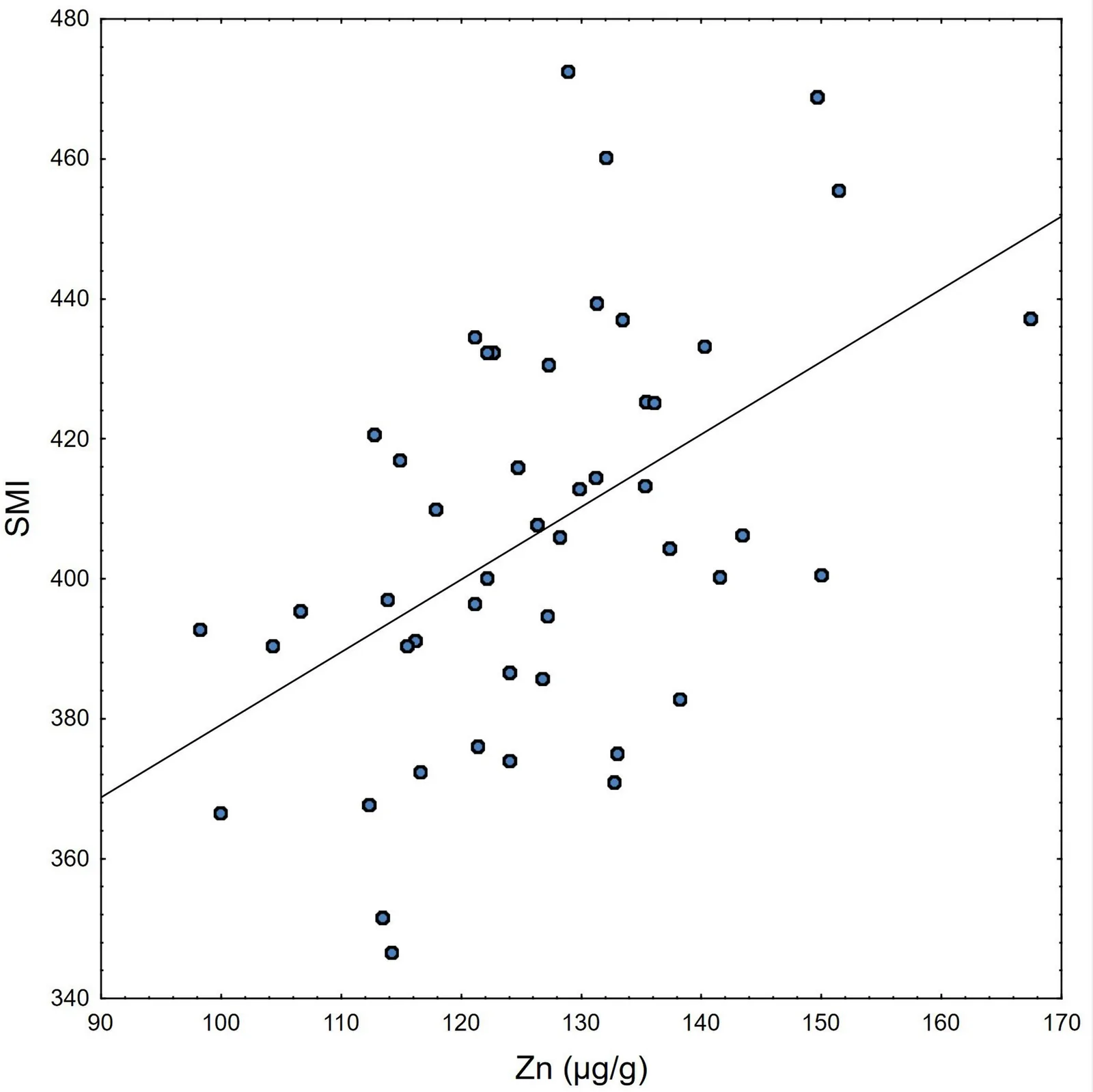
Relationship between Zn concentration in nestling feathers and their body condition expressed by the scaled mass index (SMI). The fitted regression line is shown for visualization only; the statistical relationship was tested using a GLMM; y = 1.0377 × x + 275.33, *n* = 48

Regarding feather element concentrations, older chicks exhibited higher levels of cadmium and copper compared to their younger counterparts (Fig. 2c, d), whereas zinc concentrations were higher at the earlier developmental stage (Fig. 2e). For the remaining elements, the sampling event had no significant effect on concentrations (Table 2). Simultaneously, no significant relationships were found between blood and feather concentrations of any of the analyzed elements within individuals at a given sampling event (Table 3). Furthermore, among all the elements studied in both types of tissue, only feather zinc concentration was significantly positively associated with nestling condition (Table 4, Fig. 3).

**Table 3:** Overview of GLMMs testing the relationship between element concentrations in the blood and feathers of nestlings

Element	*β*	SE	*t*	*p*
As	− 0.0210	0.0240	− 0.876	0.385
Cu	0.0001	0.0003	0.787	0.435
Pb	− 0.0260	0.0663	− 0.396	0.694
Se	0.0001	0.0002	1.882	0.066
Zn	< 0.0001	< 0.0001	− 0.028	0.977

**Table 4:** Summary of GLMMs results testing the relationships between element concentrations in nestling blood and feathers and their body condition

Sample	Element	*β*	SE	*t*	*p*
Blood	As	− 0.97	0.94	− 1.030	0.309
	Cu	− 0.04	0.03	− 1.111	0.273
	Pb	− 0.36	0.87	− 0.415	0.680
	Se	0.00	0.03	− 0.001	0.999
	Zn	0.00	0.01	− 0.451	0.654
Feathers	As	− 80.40	64.68	− 1.243	0.220
	Cd	− 70.86	335.21	− 0.211	0.834
	Cu	0.33	1.48	0.226	0.822
	Pb	2.09	16.26	0.129	0.898
	Se	16.45	9.95	1.652	0.105
	**Zn**	**0.48**	**0.20**	**2.332**	**0.024**

## Discussion

### Trace element concentrations in blood and feathers

In this study, concentrations of non-essential toxic elements were consistently low in both blood and feathers of hooded crow nestlings. Cadmium concentrations in blood were below the limit of detection, while As and Pb concentrations were low compared with values reported for other wetland-associated birds and avian predators (Tables 5 and 6; Bjedov et al., 2024; Dolan et al., 2017; Rattner et al., 2008; Sánchez-Virosta et al., 2020). These results suggest that nestlings were not exposed to high local concentrations of these elements during the period represented by blood circulation and feather growth. However, low concentrations in nestling tissues should be interpreted cautiously, because they may reflect not only low environmental contamination, but also limited bioavailability, hydrological dilution, sediment redistribution, dietary composition, and the specific exposure window represented by each biological matrix (Baborowski et al., 2007; Burger, 1993; Diehl et al., 2023; Jaspers et al., 2019; Thonon, 2006; Zduniak et al., 2008).

**Table 5:** Comparative data of element concentrations (µg/L) in the blood of nestlings and adult birds associated with freshwater wetlands and lakes, selected specialized avian predators included for contextual comparison

Age	Study areas	Species	As	Cd	Cu	Pb	Se	Zn	Method and statistics	References
Nestlings	N USA, Minnesota, pristine marshes	*Leucophaeus pipixcan*	18 ± 4	99 ± 16	–	12 ± 3	503 ± 49	–	GF-AAS, mean ± SD	Burger, 1997
	E USA, New York, Long Island	*Larus argentatus*	556 ± 77 and 166 ± 13	9 ± 1 and 47 ± 12	–	94 ± 5 and 176 ± 54	773 ± 45 and 473 ± 22	–	GF-AAS, mea*n* ± SD	Burger, 1997
	S Spain, Doñana National Park	*Ciconia ciconia*	–	–	–	–	382 (328–445)	–	ICP-MS, geometric mean (range)	Rodríquez Alvárez et al., 2013
		*Ardea purpurea*	–	–	–	–	463 (296–873)	–		
		*Plegadis falcinellus*	–	–	–	–	506 (443–656)	–		
		*Circus aeruginosus*	–	–	–	–	299 (132–487)	–		
		*Falco peregrinus*	–	–	–	–	439 (385–501)	–		
		*Milvus migrans*	–	–	–	–	293 (271–316)	–		
	S Spain, C & N Norway	*Accipiter gentilis*	1–12	0.15–0.29	230–240	4–5	350–540	4900–5500	HR-ICP-MS; range of medians	Dolan et al., 2017
	S Spain, Murcia	*Bubo Bubo*	10.0 ± 32.8	0.04 ± 0.3	225 ± 48	21.7 ± 41.8	451 ± 139	4200 ± 682	ICP-MS, mean ± SD	Sánchez-Virosta et al., 2020
Adults	N USA, Minnesota, pristine marshes	*Leucophaeus pipixcan*	5 ± 0.5	6 ± 1	–	29 ± 10	907 ± 193	–	GF-AAS; mean ± SD	Burger, 1997
	C Spain; Castilla-LaMancha, two wetlands	*Gallinula chloropus*	3.41 ± 1.05 and 4.46 ± 1.08	< LOD & 0.281 ± 0.277	455 ± 48 and 418 ± 14	6.75 ± 1.44 and 6.26 ± 0.85	425 ± 44 and 884 ± 108	6136 ± 844 and 5271 ± 134	ICP-MS; means ± SE	López-Perea et al., 2019
	N Colombia, the Magdalena River basin	*Platalea ajaja*	N/A	–	–	4.86 ± 11.11	–	–	ICP-MS; mean ± SD	Buelvas-Soto et al., 2022
		*Dendrocygna autumnalis*	20.89 ± 5.48	–	–	212.00 ± 208.10	–	–		Bjedov et al., 2024
		*Nannopterum brasilianus*	52.16 ± 50.66	–	–	46.16 ± 19.45	–	–		
	S Spain, Doñana National Park	*Ciconia ciconia*	19 (6–121)	1.5 (0.1–9.0)	586 (180–1530)	71 (2–320)	–	1900 (800–2800)	GF-AAS; mean (range)	Benito et al., 1999
		*Plegadis falcinellus*	8 (6–17)	11 (6–15)	133 (67–241)	61 (20–233)	–	900 (700–1300)		
		*Aythya ferina*	6	0.9 (0.1–3.0)	203 (151–396)	73 (25–274)	–	3700 (2500–6000)		
		*Anas platyrhynchos*	11 (6–42)	4.8 (0.1–19)	258 (142–361)	208 (45–454)	–	3300 (1300–4000)		
		*Ardea cinerea*	6	0.2 (0.1–1.0)	352 (204–650)	15 (2–89)	–	2200 (1500–3300)		
		*Anas strepera*	17 (6–29)	0.6 (0.1–29)	526 (346–753)	120 (69–174)	–	5900 (3500–8600)		
		*Platalea leucorodia*	19 (6–181)	0.3 (0.1–1.0)	307 (190–569)	8 (2–34)	–	3200 (1400–5500)		
		*Podiceps cristatus*	6	0.1	395 (327–488)	2	–	2200 (1300–2900)		
		*Larus cachinnans*	6	0.2 (0.1–0.5)	429 (271–535)	20 (9–32)	–	4400 (3100–5200)		
	S Spain, Doñana National Park	*Anas platyrhynchos*	–	–	–	–	262 (108–634)	–	ICP-MS, geometric mean (range)	Rodríquez Alvárez et al., 2013
		*Anas clypeata*	–	–	–	–	465 (335–646)	…		
		*Fulica atra*	–	–	–	–	415 (323–550)	–		
		*Bubulcus ibis*	–	–	–	–	521 (455–587)	–		
Nestlings	**W Poland, the floodplains of the Warta River**	***Corvus cornix***	**9.25 ± 3.48 (0.48); 8.70; [7.88]**	** < LOD**	**226.3 ± 111.1 (15.4); 144.8; [202.6]**	**2.94 ± 3.78 (0.52); 0.69; [1.97]**	**398.6 ± 109.6 (15.2); 383.1; [394.3]**	**2194 ± 542 (75); 2139; [2115]**	**ICP-MS; mean ± SD (SE); geometric mean; [median]**	**This study**

*HR-ICP-MS*, high resolution inductively coupled plasma mass spectrometry; *GF-AAS*, graphite furnace atomic absorption spectrometry; ICP-MS – inductively coupled plasma mass spectrometry

**Table 6:** Comparative data of element concentrations (µg/g) in feathers of nestlings, juveniles, and adult birds associated with freshwater wetlands and lakes

Age	Study areas	Species	As	Cd	Cu	Pb	Se	Zn	Method and statistics	References
Nestlings	S France, Rhone River Delta	*Phoenicopterus roseus*	0.094 ± 0.018	–	4.66 ± 0.48	0.57 ± 0.15	1.622 ± 0.309	90 ± 6	ICP-AES; mean ± SD	Borghesi et al., 2016
	S Spain, Odiel marshlands	*Phoenicopterus roseus*	0.819 ± 0.143	–	4.20 ± 0.39	0.68 ± 0.13	2.022 ± 0.209	133 ± 6	ICP-AES; mean ± SD	Borghesi et al., 2016
	NE USA, Chesapeake and Delaware Bays	*Pandion haliaetus*	0.354–1.93	0.084–0.088	4.92–9.07	0.54–1.96	0.979–1.640	71.8–100.0	ICP-MS; geometric mean	Rattner et al., 2008
	N Pakistan, Lahore area	*Bubulcus ibis*	21.4 ± 4.2	41.0 ± 1.3	52.8 ± 16.1	297 ± 11	–	529 ± 95	FAAS; geometric mean ± SE	Abdullah et al., 2015
	N Pakistan; Sialkot area	*Bubulcus ibis*	19.0 ± 2.5	37.6 ± 1.2	62.7 ± 13.6	286 ± 18.4	–	226 ± 75	FAAS; geometric mean ± SE	Abdullah et al., 2015
Juveniles	S Australia, Albert Park Lake, Melbourne	*Cygnus atratus*	–	–	11.74 ± 0.99	0.99 ± 0.15	–	133.24 ± 7.80	ICP-MS; range of means ± SE	Nzabanita et al., 2024
Adults	S Holland, floodplains of the river Waal;	*Athene noctua*	–	< 2.3	18 (14–22)	< 40	–	331 (123–1276)	ICP-AES; mean (range)	Van den Brink et al., 2003
	C Spain, Castilla-La Mancha, two wetlands	*Gallinula chloropus*	0.156 ± 0.005 and 0.378 ± 0.049	< LOD	6.52 ± 0.32 and 9.02 ± 0.52	0.036 ± 0.004 and 0.046 ± 0.009	–	71.1 ± 2.3 and 70.8 ± 3.6	ICP-MS; mean ± SE	López-Perea et al., 2019
	N Colombia, Magdalena River basin	*Platalea ajaja*	0.05 ± 0.02	–	–	0.12 ± 0.10	–	–	ICP-MS; mean ± SD	Buelvas-Soto et al., 2022
		*Dendrocygna autumnalis*	0.71 ± 0.57	–	–	0.87 ± 0.41	–	–		Bjedov et al., 2024;
		*Nannopterum brasilianus*	2.40 ± 0.89	–	–	7.40 ± 0.51	–	–		
	N Pakistan, the Baroghil River valley	*Aythya ferina*	–	0.30 ± 0.09	1.84 ± 0.56	0.91 ± 0.03	–	52.32 ± 4.41	FAAS; mean ± SD	Abbasi et al., 2015
		*Anas crecca*	–	0.53 ± 0.13	1.96 ± 0.65	1.19 ± 0.74	–	46.72 ± 9.55		
		*Anas acuta*	–	0.68 ± 0.16	2.26 ± 0.66	0.98 ± 0.37	–	54.38 ± 5.98		
	N Pakistan, the Soan River valley	*Aythya ferina*	–	0.75 ± 0.26	2.23 ± 1.28	1.97 ± 0.54	–	87.67 ± 14.04	FAAS; mean ± SD	Abbasi et al., 2015
		*Anas crecca*	–	1.06 ± 0.23	3.19 ± 0.31	2.34 ± 0.31	–	61.00 ± 5.33		
		*Anas acuta*	–	1.05 ± 0.06	3.90 ± 0.38	2.64 ± 0.30	–	73.74 ± 11.82		
	W Hungary, Lake Balaton	*Cygnus olor*	< 0.5	0.5	10.24 ± 2.25	1.11 ± 1.23	–	–	ICP-OES; mean ± SD	Grúz et al.m 2015
	S India, Bellandur lake	*Corvus splendens*	–	3.41 ± 1.39	–	24.6 ± 6.68	–	–	ICP-MS; mean ± SD	Kumar & Chandrahasan, 2023
Nestlings	**W Poland, the floodplains of the Warta River**	***Corvus cornix***	**0.082 ± 0.049 (0.007)**	**0.004 ± 0.009 (0.001)**	**4.51 ± 1.95 (0.27)**	**0.14 ± 0.17 (0.02)**	**1.718 ± 0.265 (0.036)**	**126.77 ± 13.17 (1.80)**	**ICP-MS; mean ± SD (SE)**	**This study**

*FAAS*, flame atomic absorption spectrometry; *ICP-AES*, inductively coupled plasma atomic emission spectrometry; *ICP-OES*, inductively coupled plasma optical emission spectrometry; *ICP-MS*, inductively coupled plasma mass spectrometry

The study area is a dynamic floodplain system influenced by seasonal water-level fluctuations, sediment transport, and hydrological mixing between the Warta River, local water bodies, and tributaries (Choiński, 2000; Zduniak, 2009). Although large lowland rivers may transport contaminants from extensive catchments (Jaskuła et al., 2021; Krodkiewska et al., 2022), exposure of organisms inhabiting floodplains depends strongly on local processes operating within floodplain habitats, including sediment deposition, redistribution, dilution, and hydrological dynamics (Baborowski et al., 2007; Diehl et al., 2023; Thonon, 2006). Flooding may introduce contaminated suspended matter, but it may also dilute pollutants, redistribute sediments, and influence the local bioavailability of some elements. In addition, the inflow of relatively less polluted water from the Postomia River may contribute to spatial variation in exposure within the study area (Siepak et al., 2023). Therefore, the low concentrations of Cd, Pb, and As observed in hooded crow nestlings may indicate limited local transfer of these elements into the food web used by this species, rather than simply the absence of potential pollution sources in the wider catchment.

The omnivorous diet of hooded crow nestlings should also be considered when interpreting these results. In wetland habitats, nestlings may receive food items from aquatic, semi-aquatic, and terrestrial compartments, including fish, molluscs, crayfish, amphibians, eggs, nestlings, carrion, and other animal material (Zduniak et al., 2008). Such a broad trophic niche may buffer exposure to contaminants if highly contaminated food items represent only a limited part of the diet. At the same time, this ecological flexibility makes the hooded crow a useful sentinel species for assessing local exposure across multiple trophic pathways. However, because hooded crows are omnivorous generalists, comparisons with specialized avian predators should be treated as contextual rather than as evidence of equivalent trophic position or biomagnification potential.

The interpretation of low concentrations should also consider the biological meaning of the analyzed matrices. The blood generally represents recent or current exposure, whereas feathers integrate exposure during feather growth and may reflect tissue-specific deposition of elements (Burger, 1993; Jaspers et al., 2019; Tsipoura et al., 2017). Therefore, low blood concentrations of Cd, Pb, and As suggest limited recent exposure, while low feather concentrations suggest limited deposition during feather formation. Taken together, the absence of elevated concentrations in either matrix indicates that exposure to non-essential toxic elements was probably low during the nestling period examined in this study.

For essential elements, Cu, Se, and Zn concentrations should be interpreted differently because these elements are physiologically regulated and involved in growth, antioxidant defence, immune function, feather development, and pigmentation (Beck & Hopkins, 2015; Galván & Solano, 2016; Giraudeau et al., 2015; Koim-Puchowska et al., 2020). Their concentrations in the blood and feathers may therefore reflect both environmental availability and developmental requirements. In particular, relatively high Zn concentrations in feathers may be related to the role of Zn in feather formation and melanin-based pigmentation, especially in dark feathers (Chatelain et al., 2016; Hanć et al., 2017). Thus, variation in essential element concentrations should not be interpreted solely as environmental contamination, but also as a result of physiological regulation and tissue-specific allocation during rapid nestling growth.

### Age-related concentrations

We observed age-related differences in the concentrations of several elements in the blood and feathers of hooded crow nestlings. Blood Cu and Se concentrations were higher in older nestlings than during the earlier sampling event. This pattern may be associated with developmental changes during the nestling period, as both elements are involved in antioxidant defence, immune function, and growth-related physiological processes (Beck & Hopkins, 2015; Koim-Puchowska et al., 2020). However, age-related variation in element concentrations should not be interpreted solely as a direct increase in physiological demand. During rapid nestling development, increasing body mass and tissue growth may also dilute the concentration of some elements, while changes in diet, parental provisioning, metabolism, and tissue allocation may alter circulating element levels.

In feathers, older nestlings had higher Cu and Cd concentrations, whereas Zn concentrations were higher at the earlier developmental stage. These patterns should be interpreted in relation to feather ontogeny, because feathers collected during the first and second sampling events represented different stages of development. The first sampling coincided with the early phase of primary feather growth, whereas the interval between sampling events was associated mainly with feather elongation and degradation of the pin sheath. Therefore, differences in feather element concentrations may partly reflect changes in growth rate, keratinization, and element deposition, rather than only changes in whole-body burden.

The higher Cu concentration in feathers of older nestlings may reflect tissue-specific deposition, in parallel with increased blood Cu concentrations. Copper is involved in feather structure and pigmentation processes, and its allocation to growing feathers may vary during development (Galván & Solano, 2016; Giraudeau et al., 2015). By contrast, the decrease in feather Zn concentration between sampling events may be related to higher Zn requirements during the early phase of feather development. Zinc concentrations in feathers were much higher than Cu concentrations, and Zn is associated with feather formation and melanin-based pigmentation, particularly in dark feathers (Chatelain et al., 2016; Hanć et al., 2017).

Among the non-essential toxic elements, only Cd showed a significant age-related increase in feathers, while its concentration in blood remained below the limit of detection. This pattern may be consistent with deposition of Cd into growing feathers, which can act as one route of element sequestration or elimination in birds (Burger, 1993; Chatelain et al., 2014). However, because feathers were sampled at different stages of development, this result should be interpreted cautiously. The increase in feather Cd may reflect not only possible detoxification-related deposition, but also feather ontogeny, tissue-specific binding, redistribution during growth, and element-specific toxicokinetics. Growth or mass dilution may additionally influence age-related concentrations in blood and other tissues during rapid nestling development, as suggested by other studies of trace element dynamics in growing birds (Orłowski et al., 2024).

### Blood–feather relationships

We found no significant relationships between blood and feather concentrations of the analyzed elements in hooded crow nestlings. This result is not unexpected, because blood and feathers represent different biological matrices and different temporal windows of exposure. Blood concentrations generally reflect recent or current exposure, whereas feathers integrate element deposition during feather growth (Burger, 1993; Jaspers et al., 2019; Tsipoura et al., 2017). In developing nestlings, these relationships may be further modified by rapid growth, changes in diet and parental provisioning, physiological regulation of essential elements, and the developmental stage of feathers at the time of sampling.

The absence of significant blood–feather relationships may also be related to the low concentrations of non-essential toxic elements recorded in this study. When exposure levels are low, variation in the blood and feather concentrations may be too small or too strongly affected by tissue-specific processes to produce clear relationships between matrices. In addition, element-specific binding affinities and heterogeneous distribution among tissues may contribute to differences between the blood and feather concentrations (Ding et al., 2023; Orłowski et al., 2012; Rodríguez-Álvarez et al., 2022).

Previous studies have shown that relationships between blood and feather concentrations are not uniform across bird species or elements. For example, weak or moderate associations have been reported for some elements in Ospreys and Northern Goshawks, whereas other studies on wetland birds found no consistent relationships between the two matrices (Bjedov et al., 2024; Dolan et al., 2017; Rattner et al., 2008). Such variation probably reflects interspecific differences in diet, trophic ecology, exposure level, metabolism, feather growth, and local environmental contamination. Therefore, the lack of significant relationships in hooded crow nestlings suggests that the blood and feathers should be treated as complementary, rather than interchangeable, matrices in biomonitoring studies of developing birds.

### Element concentrations and body condition

Among all analyzed elements, only Zn concentration in feathers was positively associated with nestling body condition. This relationship may reflect the physiological role of Zn during nestling development, because Zn is involved in growth, feather formation, skeletal and muscular development, and pigmentation processes (Chatelain et al., 2016; Hanć et al., 2017; Jennings & Winkler, 2020; Gopi et al., 2025). In our study, feather Zn concentrations were moderate compared with values reported for other wetland-associated birds, suggesting that the observed relationship was unlikely to reflect excessive Zn exposure. Instead, higher feather Zn concentrations may indicate better nutritional status, more efficient allocation of essential elements to growing feathers, or differences in feather development among nestlings.

The lack of significant relationships between Cd, As, and Pb concentrations and nestling condition should be interpreted cautiously. Concentrations of these non-essential toxic elements were low in both blood and feathers, and Cd in the blood was below the limit of detection. Therefore, the absence of detectable associations with body condition may reflect low exposure levels, limited statistical variation in element concentrations, or the fact that SMI may not capture subtle physiological effects. Because toxic effects were not directly assessed in this study, our results do not demonstrate an absence of toxicity, but rather indicate that no relationship between measured concentrations of non-essential toxic elements and nestling body condition was detected under the observed exposure levels.

## Conclusions

Our results indicate low concentrations of non-essential toxic elements, including Cd, As, and Pb, in the blood and feathers of hooded crow nestlings from the studied floodplain wetland. This suggests limited local exposure and/or low bioavailability of these elements in the food web used by this omnivorous, wetland-associated species during the nestling period. However, these findings should not be interpreted as a complete assessment of the ecological status of the ecosystem, but rather as evidence of low trace element exposure in the sampled nestlings and tissues.

We also found age-related variation in the concentrations of essential elements, including Cu, Se, and Zn. Blood Cu and Se concentrations were higher in older nestlings, while feather Cu and Cd concentrations increased between sampling events, and feather Zn concentrations were higher at the earlier developmental stage. These patterns probably reflect a combination of physiological regulation, growth requirements, tissue-specific deposition, feather development, and element-specific toxicokinetics. Therefore, nestling age and feather growth stage should be carefully considered when interpreting trace element concentrations in developing birds.

Blood and feather concentrations were not significantly related, indicating that these matrices provide complementary rather than interchangeable information on exposure. Feather Zn concentration was positively associated with nestling body condition, whereas no significant relationships were detected between Cd, As, or Pb concentrations and body condition under the observed low exposure levels. Overall, this study highlights the value of combining blood and feather analyses in avian biomonitoring, while also emphasising the need to account for tissue type, nestling development, and local floodplain processes when interpreting trace element data.

## Data Availability

The data that support the findings of this study are available from the corresponding author upon reasonable request.
